# Moderating role of coping in the association between minority stress and suicidal ideation and suicide attempts among sexual and gender minority young adults

**DOI:** 10.1111/sltb.12913

**Published:** 2022-09-02

**Authors:** Jennifer de Lange, Laura Baams, Henny Bos, Roel Bosker, Eva Dumon, Gwendolyn Portzky, Jo Robinson, Diana van Bergen

**Affiliations:** ^1^ Department of Pedagogy and Educational Sciences University of Groningen Groningen The Netherlands; ^2^ Department of Child Development and Education University of Amsterdam Amsterdam The Netherlands; ^3^ Department of Head and Skin, Flemish Centre of Expertise in Suicide Prevention Ghent University Ghent Belgium; ^4^ Orygen Parkville Victoria Australia; ^5^ Centre for Youth Mental Health University of Melbourne Parkville Victoria Australia

**Keywords:** coping, minority stress, sexual and gender minority, suicidality, young adults

## Abstract

**Objective:**

This study examined associations of minority stressors (i.e., victimization, internalized homonegativity, and stigma consciousness), and coping styles (i.e., active, avoidant, and passive) with suicidal ideation and suicide attempts (suicidality) among sexual and gender minority (SGM) young adults, and whether coping style moderated these associations.

**Methods:**

Logistic regression analyses examined these associations among 1432 SGM young adults (ages 18–29).

**Results:**

Minority stressors and passive coping were associated with a higher likelihood of suicidality. Avoidant coping was associated with a lower likelihood of lifetime suicidal ideation and attempts among sexual minority participants, and active coping with a lower likelihood of past‐year suicidal ideation among sexual minority participants. Moderation analyses among sexual minority participants showed that when avoidant coping was high, associations between low victimization (compared with no victimization) and lifetime suicide attempts, and stigma consciousness and lifetime suicide attempts became non‐significant, and the association between internalized homonegativity and lifetime suicide attempts became significant. Among gender minority participants, when passive coping was high the association between low victimization and lifetime suicidal ideation became significant.

**Conclusion:**

This study underlines the importance of minority stress and coping for suicidality among SGM young adults and the need for more research regarding the role of coping.

## INTRODUCTION

The rates of suicidal ideation and suicide attempts among sexual and gender minority (SGM) young adults are higher than among heterosexual, cisgender young adults (Salway et al., 2021; Williams et al., 2021). Mental health disparities for SGM individuals are often explained using constructs from the minority stress framework (Meyer, 2003) and extensions hereof (Hatzenbuehler, 2009; Hendricks & Testa, 2012). The literature posits that SGM individuals may experience stress related to their minority identity and that excess stress explains disparities in mental health (Meyer, 2003; Testa et al., 2015), such as suicidal ideation and attempts (suicidality) (de Lange et al., 2022; Hatchel et al., 2019; Testa et al., 2017; Williams et al., 2021). Research among SGM young people showed that stressors such as victimization, rejection, perceived stigma, and internalized homonegativity or transnegativity were the risk factors for suicidal ideation or attempts (Kaniuka et al., 2019; Kuper et al., 2018; Lea et al., 2014; Mustanski & Liu, 2013; Ryan et al., 2009).

There are considerable individual differences in the impact of minority stress on suicidality; particularly how individuals cope with minority stress are relevant. However, limited research has been conducted among SGM young adults on the role of coping in the association between minority stress and suicidality. The current study examines links between minority stress, coping styles, and suicidal ideation and attempts among SGM young adults and whether coping styles moderate the associations between minority stress and suicidal ideation and attempts.

### Coping and suicidality among SGM adolescents and young adults

Coping has been described as “action regulation under stress.” Action regulation refers to “efforts to mobilize, manage, and direct physiology, emotion, attention, behavior and cognition in response to stress” (Skinner & Zimmer‐Gembeck, 2007, p. 123). Research suggests that passive and avoidant coping strategies are related to *higher* levels of suicidal ideation (Benatov et al., 2020; Ong & Thompson, 2019), while active and problem‐oriented strategies are related to *lower* levels of suicidal ideation (Benatov et al., 2020; Speckens & Hawton, 2005).

To the best of our knowledge, no studies examined associations between coping and suicidality among SGM individuals; however, several studies examined coping and other mental health outcomes among SGM individuals. For example, qualitative research showed that (ethnically diverse) SGM youth might use avoidant coping strategies such as hiding their sexual orientation or gender identity (Goldbach & Gibbs, 2015; Kuper et al., 2014; van Bergen & Spiegel, 2014), or use direct problem‐solving to manage minority stress (van Bergen & Spiegel, 2014). In addition, quantitative research demonstrated that avoidant coping (Bos et al., 2014; Budge et al., 2013), palliative reactions (e.g. seeking distraction), passive reactions (e.g., brooding) (Bos et al., 2014), cognitive strategies (e.g., distraction), alternative‐seeking strategies (Toomey et al., 2018), emotion‐oriented coping strategies (Grossman et al., 2011), and maladaptive coping (e.g., such as self‐destruction and denial) (Lehavot, 2012) were all associated with poorer mental health among SGM individuals, including anxiety, psychological distress, and depressive symptoms.

Studies have also identified coping strategies that are related to *better* mental health outcomes. For example, LGBT‐specific coping (e.g., “looking for information on LGBT issues”) (Toomey et al., 2018), active coping, and problem‐oriented coping (e.g., active problem‐solving) (Bos et al., 2014; D'haese et al., 2016), and adaptive coping (such as planning and acceptance) (Lehavot, 2012) were all associated with better outcomes, including less depressive symptoms and lower psychological distress. Further, contrary to the findings of other studies (Bos et al., 2014; Budge et al., 2013), avoidant coping was associated with *better* mental health outcomes among lesbian, gay, and bisexual (LGB) individuals (D'haese et al., 2016).

Coping may also mitigate or enhance the impact of minority stress on mental health (Meyer, 2003). In line with this, the integrated motivational‐volitional model of suicidal behavior (IVM) theorizes that coping responses may affect the feelings of entrapment, which subsequently may influence suicidal ideation (O'Connor & Kirtley, 2018). To the best of our knowledge, there is currently no study among SGM young adults on the strength of the association between minority stressors and suicidality by coping strategy. Nevertheless, some studies assessed the role of coping in the association between minority stress and mental health. For example, one study among LGB adults showed that emotion‐oriented coping, problem‐oriented coping, or avoidant coping did not moderate the relation between homophobic violence and mental health (D'haese et al., 2016). Similarly, studies among sexual minority individuals found no moderating effect of avoidant coping on the association of heterosexist events and psychological distress among men (Szymanski, 2009), and no moderating effect of avoidant or problem‐solving coping on the association between internalized heterosexism and psychological distress among women (Szymanski & Owens, 2008). Another study, among sexual minority young people, found that problem‐solving coping skills did not moderate the association between minority stressors and depression with perceived burdensomeness and thwarted belongingness as mediators (Baams et al., 2018).

### Current study

In the current study, we examined to what extent different minority stressors (e.g., internalized homonegativity, stigma consciousness, and victimization) were associated with suicidality among SGM young adults. Based on the prior research (de Lange et al., 2022; Hatchel et al., 2019; Testa et al., 2017; Williams et al., 2021), we hypothesized minority stressors to be associated with a higher likelihood of suicidal ideation and attempts. In addition, we examined to what extent active, avoidant, and passive coping styles were associated with suicidal ideation and attempts. Based on the previous findings (Benatov et al., 2020; Bos et al., 2014; Budge et al., 2013; Ong & Thompson, 2019; Speckens & Hawton, 2005), we hypothesized active coping to be associated with a lower likelihood of suicidal ideation and attempts, and avoidant coping and passive coping to be associated with a higher likelihood of suicidal ideation and attempts. Last, we explored whether these coping styles moderated the associations between minority stressors and suicidal ideation and attempts.

## METHOD

### Procedure and participants

This study used data from 2625 SGM participants aged between 18 and 80 years old from Flanders (Belgium) (collected September 2015–March 2016), and the Netherlands (collected October 2016–February 2017). All participants completed online questionnaires in Dutch. Participants were recruited online via social media and LGBT organizations, and two suicide prevention websites in Flanders and the Netherlands; snowballing techniques were also used. Additionally, flyers advertising the study were distributed to LGBT and mental health organizations and distributed at LGBT events.

For the present study, data from SGM young adults between 18 and 29 years were included, resulting in a sample of *N* = 1451 SGM young adults. Data from 19 participants who identified as “man or woman who engages in cross‐dressing” were excluded, because it was not clear whether they (also) self‐identified as transgender or genderqueer. The final sample consisted of *N* = 1127 cisgender sexual minority young adults between ages 18 and 29 (M = 22.12, SD = 3.20) and *N* = 305 gender minority young adults between ages 18 and 29 (M = 21.76, SD = 3.21). See Tables 1 and 2 for sample demographics.

**TABLE 1:** Sample characteristics

	Sexual minority young adults (*n* = 1127) (%)	Gender minority young adults (*n* = 305) (%)
Lifetime suicidal ideation	60.6	82.0
Past‐year suicidal ideation	28.9	49.8
Lifetime suicide attempts	17.2	36.1
Past‐year suicide attempts	3.5	10.2
Sex assigned at birth		
Female	61.6	78.4
Male	38.4	21.6
Gender identity		
Cisgender	100	
Trans man	–	27.9
Trans woman	–	12.1
Genderqueer	–	51.8
Other^a^	–	8.2
Education^b^		
Lower	23.5	39.7
Higher	76.5	60.3
Country of residence		
Belgium	35.4	36.1
The Netherlands	64.6	63.9

^a^ For example, demigender, agender, or not sure.

^b^ Educational level was assessed as high or low, depending on whether they had completed college or university or not.

**TABLE 2:** Sexual attraction by gender identity

	Cisgender woman (*n*)	Cisgender man (*n*)	Transgender man (*n*)	Transgender woman (*n*)	Gender queer (*n*)	Other (*n*)
Only to women	30.3% (210)	0% (0)	18.8% (16)	5.4% (2)	20.3% (32)	12% (3)
Mostly to women, rarely to men	31.8% (221)	0.9% (4)	18.8% (16)	27.0% (10)	22.3% (35)	20% (5)
A little more to women than to men	14.1% (98)	0.5% (2)	3.5% (3)	2.7% (1)	6.3% (10)	4% (1)
Equally to men and women	8.2% (57)	1.8% (8)	4.7% (4)	10.8% (4)	3.2% (5)	8% (2)
A little more to men than to women	6.8% (47)	2.8% (12)	10.6% (9)	0% (0)	5.7% (9)	8% (2)
Mostly to men, rarely to women	1.0% (7)	25.9% (112)	8.2% (7)	2.7% (1)	7.0% (11)	8% (2)
Only to men	0% (0)	65.8% (285)	11.8% (10)	18.9% (7)	3.8% (6)	8% (2)
Neither	0.9% (6)	0% (0)	4.7% (4)	8.1% (3)	5.1% (8)	8% (2)
Gender (man or woman) is not important to me	6.3% (44)	2.1% (9)	17.6% (15)	21.6% (8)	24.7% (39)	20% (5)
I do not know	0.6% (4)	0.2% (1)	1.2% (1)	2.7% (1)	1.9% (3)	4% (1)

Ethics approval was given by the ethics committee of the Department of Pedagogy and Educational Sciences of the University of Groningen, and the Ghent University Hospital. All participants provided informed consent.

### Measures

#### Suicidality

Lifetime suicidal ideation was assessed with the question: “Have you ever seriously thought about ending your life?” Response options “yes, multiple times,” “yes, once,” and, “no, never” were dichotomized into yes (1) and no (0). When a participant answered yes, they received the follow‐up question: “Have you had these [suicidal] thoughts during the past 12 months?” Answer options were “yes” (1) and “no” (0). Participants who answered *no* to lifetime suicidal ideation were also assigned (0).

Lifetime suicide attempts was assessed with the question “Have you ever attempted suicide?” Response options “yes, multiple times,” “yes, once,” and “no, never” were dichotomized into yes (1) and no (0). When a participant answered yes, they received the follow‐up question: “Have you attempted suicide in the past 12 months?” Answer options were “yes” (1) and “no” (0). Participants who answered *no* to lifetime suicide attempts were also assigned (0).

#### Minority stressors

Internalized homonegativity was assessed with a nine‐item scale (Cronbach's α = 0.78) derived from the Internalized Homonegativity Inventory (Mayfield, 2001). An example item is: “I sometimes feel embarrassed because of my sexual orientation.” The scale ranged from 1 (*strongly disagree*) to 5 (*strongly agree*). Internalized homonegativity is only included in the analyses for the sexual minority group.

LGB and trans‐related victimization was assessed with the single item: “Have you ever been a victim of homophobic or transphobic violence?” Response options were: (1) never, (2) once in a while, (3) about once a month, (4) several times a month, (5) about once a week, (6), several times a week, and (7) daily. This was categorized into never (1), low (2), high (3 through 7).

Stigma consciousness was assessed with the stigma consciousness questionnaire (SCQ; Pinel, 1999). This questionnaire consists of 10 items (sexual minority (SM) sample Cronbach's α = 0.74; gender minority (GM) sample Cronbach's α = 0.80). The questionnaire was adapted for gender identity to apply to experiences of gender minority individuals. For example, “I never worry that my behavior will be viewed as stereotypical for transgender individuals.” The scale ranged from 1 (*strongly disagree*) to 5 (*strongly agree*).

#### Coping

Coping was assessed with The Utrecht Coping List (UCL) (Schreurs et al., 1993). For the current study, we included three coping styles that based on the literature were most likely to be related to suicidality (Benatov et al., 2020; Ong & Thompson, 2019; Speckens & Hawton, 2005): active, avoidant, and passive coping. Active coping consisted of six items (SM Cronbach's α = 0.83; GM Cronbach's α = 0.82) and refers to actively approaching the problem to solve it. An item in this scale is: “think of different possibilities to solve a problem.” Avoidant coping consisted of eight items, however, after a reliability analysis, it was decided to delete one item from this scale (SM Cronbach's α = 0.74; GM Cronbach's α = 0.78) and refers to avoiding situations. An item in this scale is: “avoiding difficult situations as much as possible.” Passive coping consisted of seven items (SM Cronbach's α = 0.80; GM Cronbach's α = 0.77) and refers to brooding and feeling like not being able to cope. An item in this scale is: “not feeling capable to do something.” The scale ranged from 1 (*rarely or never*) to 4 (*very often*).

#### Sexual orientation and gender identity

Sexual orientation was assessed with the question “Throughout your life, who are you sexually attracted to?” Response options were “only to women,” “mostly to women, rarely to men,” “a little more to women than to men,” “equally to men and women,” “a little more to men than to women,” “mostly to men, rarely to women,” “only to men,” “neither,” “gender (man or woman) is not important to me,” “I do not know.”

Gender identity was assessed with the question “For some people, sex assigned at birth does not (fully) match their identity as male or female. Could you tell us how you currently identify?” Answer categories were “man,” “woman,” “man who cross‐dresses,” “woman who cross‐dresses,” “trans man,” “trans woman,” “genderqueer, poly gender, or gender fluid,” “different, namely.” Participants who stated to be assigned male at birth and identified as men, and participants who stated to be assigned female at birth and identified as women, were recoded into cisgender and not included in the gender minority models.

#### Demographics

Sex assigned at birth was assessed with the question “At birth, you were assigned as:” Answer options were “male” and “female.” Age was asked in number of years. Participants' educational level was assessed as *high* or *low*, depending on whether they had completed college or university or not. Country was assessed by the question in which country the participants lived at the moment of filling out the questionnaire (*Belgium* or *the Netherlands*). Data from participants not residing in one of those two countries were removed from the analyses.

### Analysis

The data of the included participants were screened for duplicate observations, and open text fields were screened for mock answers. In addition, the data were assessed for outliers and response patterns.

Analyses were conducted using IBM SPSS Statistics (version 26). Three outcomes were assessed in logistic regression analyses: lifetime suicidal ideation, lifetime suicide attempts, and past‐year suicidal ideation. To assess the independent associations between coping styles, minority stress, and suicidality, we ran one model including all minority stressors and one model including coping styles (active, avoidant, and passive coping). To gain insight into the associations between coping styles and suicidality, we added the coping styles in three steps. The first step included age, sex assigned at birth, and active coping. In the second step, we added avoidant coping, and in the third step, we added passive coping. Minority stressors included victimization, stigma consciousness, and internalized homonegativity (only for sexual minority participants). Interaction effects between the minority stressors and coping style were added to the models, along with minority stressors and coping style. To probe significant interaction effects, simple slopes analyses (Hayes, 2018) were conducted. Age and sex assigned at birth were added as control variables in all models because both were significantly correlated with key study variables (see Table 3).

**TABLE 3:** Correlations and descriptives for key variables

	Sexual minority young adults		Gender minority young adults		1	2	3	4	5	6	7	8
	*n*	M (SD)	*n*	M (SD)								
1. Age	1127	22.12 (3.20)	305	21.76 (3.21)	–	0.17**	0.00	0.06	n/a	0.12*	−0.04	−0.06
2. Sex assigned at birth^a^					0.15**	–	–	0.01	n/a	−0.10	−0.00	0.14*
3. Victimization^b^					0.03	–	–	0.12*	n/a	0.00	0.01	0.11
4. Stigma	1123	2.77 (0.59)	269	3.19 (0.68)	0.02	0.13**	0.29**	–	n/a	−0.12*	0.24**	0.26**
5. Internalized homonegativity	1123	2.23 (0.61)	–	–	−0.07*	0.01	−0.05	0.32**	–	n/a	n/a	n/a
6. Active coping	1127	2.43 (0.62)	305	2.29 (0.60)	0.14**	0.14**	−0.06*	−0.14**	−0.23**	–	−0.24**	−0.44**
7. Avoidant coping	1127	2.26 (0.52)	305	2.39 (0.59)	−0.08**	−0.07*	−0.02	0.11**	0.23**	−0.33*	–	0.55**
8. Passive coping	1127	2.23 (0.64)	305	2.51 (0.64)	−0.11**	−0.11**	0.13**	0.28**	0.28**	−0.52**	0.50**	–

*Note*: Sex assigned at birth (0 = female; 1 = male); victimization (1 = never; 2 = low; 3 = high). Above the diagonal correlations for the gender minority sample are shown and below the diagonal correlations for the sexual minority sample are shown. N/a = not applicable; internalized homonegativity was not assessed among gender minority participants.

* *p* < 0.05.

** *p* < 0.01.

^a^ Point biserial correlation.

^b^ Spearman's correlation (all other correlations were Pearson's).

Because of too few events in past‐year suicide attempts, the associations between minority stressors, coping styles, and past‐year suicide attempts were assessed with multi‐ and uni‐variate analyses. manova analyses were conducted for differences in stigma, internalized homonegativity (only sexual minority participants), active, avoidant, and passive coping by past‐year suicide attempt. Further, Chi‐Square tests were performed to assess the association between victimization and past‐year suicide attempts. Post hoc pairwise comparisons with Bonferroni correction were utilized to assess which groups differed from another.

## RESULTS

### Descriptives

Correlations between key variables are shown in Table 3.

### Associations between minority stressors and suicidality

Table 4 shows the results of the logistic regression analyses with minority stressors as independent variables. For sexual minority young adults, results showed that compared with no victimization, participants who reported low victimization or high victimization were more likely to report lifetime suicidal ideation and lifetime suicide attempts. Victimization was not associated with past‐year suicidal ideation. Higher levels of internalized homonegativity were associated with higher odds of lifetime and past‐year suicidal ideation. Internalized homonegativity was not associated with lifetime suicide attempts. Further, participants who reported higher levels of stigma consciousness were more likely to report lifetime suicidal ideation, lifetime suicide attempts, and past‐year suicidal ideation.

**TABLE 4:** Results of logistic regression analyses of minority stressors and suicidality

	Lifetime suicidal ideation	Lifetime suicide attempts	Past‐year suicidal ideation
	aOR [95% CI]	aOR [95% CI]	aOR [95% CI]
**Sexual minority young adults**			
Victimization (ref = never)			
Low	**1.92 [1.46, 2.53]**	**1.83 [1.24, 2.70]**	1.14 [0.84, 1.55]
High	**2.28 [1.50, 3.47]**	**3.41 [2.11, 5.53]**	1.54 [1.00, 2.36]
Internalized homonegativity	**1.53 [1.22, 1.92]**	1.10 [0.84, 1.44]	**2.38 [1.87, 3.02]**
Stigma consciousness	**1.78 [1.39, 2.26]**	**1.58 [1.17, 2.13]**	**1.50 [1.16, 1.95]**
**Gender minority young adults**			
Victimization (ref = never)			
Low	**3.88 [1.72, 8.77]**	1.87 [0.89, 3.93]	**2.16 [1.08, 4.32]**
High	**2.82 [1.18, 6.73]**	**3.57 [1.64, 7.76]**	**2.34 [1.11, 4.96]**
Stigma consciousness	1.34 [0.79, 2.25]	1.26 [0.86, 1.84]	**2.15 [1.43, 3.25]**

*Note*: Controlling for sex assigned at birth and age. Bold estimates are significant, *p* < 0.05.

Abbreviations: aOR, adjusted odds ratio; CI, confidence interval; Low victimization, sometimes; High victimization, once per month or more.

For gender minority young adults, results showed that compared with no victimization, participants who reported low victimization or high victimization were more likely to report lifetime and past‐year suicidal ideation. High victimization was also associated with higher odds of lifetime suicide attempts. In addition, participants who reported higher levels of stigma consciousness were more likely to report past‐year suicidal ideation. Stigma consciousness was not associated with lifetime suicidal ideation and suicide attempts.

Regarding past‐year suicide attempts, the results of the manova analyses and Chi‐Square tests are shown in Table 5. Sexual minority and gender minority participants who reported past‐year suicide attempts had a higher mean level of stigma consciousness than participants who did not report past‐year suicide attempts. Sexual minority participants who reported past‐year suicide attempts reported higher levels of internalized homonegativity. For sexual minority participants, a Chi‐Square test demonstrated that victimization was significantly associated with past‐year suicide attempts. Sexual minority participants who reported high victimization reported past‐year suicide attempts more often than sexual minority participants who reported no victimization. For gender minority participants, victimization was not significantly associated with past‐year suicide attempts.

**TABLE 5:** Results of manova analyses and Chi‐square tests for past‐year suicide attempts

	Past‐year suicide attempt no	Past‐year suicide attempt yes			
**Sexual minority young adults**					
Victimization			*χ* ^2^ (2, *N* = 1127) = 19.10, *p* < 0.001		
Never	98.2% (*n* = 436)	1.8% (*n* = 8)			
Low	96.9% (*n* = 500)	3.1% (*n* = 16)			
High	91.0% (*n* = 152)	9.0% (*n* = 15)			
	M (SD)	M (SD)	*df*	*F*	*p*
	*n* = 1084	*n* = 39			
Internalized homonegativity	2.22 (0.61)	2.61 (0.72)	1121	15.36	<0.001
Stigma consciousness	2.76 (0.59)	3.11 (0.68)	1121	13.85	<0.001
Active coping	2.44 (0.61)	1.94 (0.61)	1121	25.77	<0.001
Avoidant coping	2.25 (0.52)	2.46 (0.56)	1121	5.73	0.017
Passive coping	2.20 (0.63)	2.83 (0.67)	1121	36.90	<0.001
**Gender minority young adults**					
Victimization			*χ* ^2^ (2, *N* = 305) = 5.36, *p* = 0.069		
Never	93.7% (*n* = 59)	6.3% (*n* = 4)			
Low	91.9% (*n* = 137)	8.1% (*n* = 12)			
High	83.9% (*n* = 78)	16.1% (*n* = 15)			
	M (SD)	M (SD)	*df*	*F*	*p*
	*n* = 243	*n* = 26			
Stigma consciousness	3.14 (0.66)	3.67 (0.72)	267	15.31	<0.001
Active coping	2.33 (0.58)	1.98 (0.68)	267	8.18	0.005
Avoidant coping	2.41 (0.59)	2.37 (0.55)	267	0.15	0.702
Passive coping	2.48 (0.63)	2.78 (0.65)	267	5.39	0.021

Abbreviations: M, mean; SD, standard deviation.

### Associations between coping and suicidality

Table 6 shows the results of the stepwise regression analysis with coping styles as independent variables. The results from step 1, which included active coping, showed that for both sexual minority and gender minority participants, higher levels of active coping were associated with a lower likelihood of lifetime and past‐year suicidal ideation and lifetime suicide attempts.

**TABLE 6:** Logistic regression analyses of coping styles and suicidality

	Model 1	Model 2	Model 3
	aOR [95% CI]	aOR [95% CI]	aOR [95% CI]
**Lifetime suicidal ideation**			
**Sexual minority young adults**			
Active coping	**0.44 [0.36, 0.55]**	**0.49 [0.39, 0.61]**	0.97 [0.74, 1.25]
Avoidant coping		**1.48 [1.15, 1.91]**	**0.65 [0.48, 0.88]**
Passive coping			**7.55 [5.47, 10.44]**
**Gender minority young adults**			
Active coping	**0.56 [0.34, 0.92]**	0.64 [0.38, 1.08]	0.86 [0.49, 1.53]
Avoidant coping		1.71 [0.99, 2.94]	1.07 [0.56, 2.04]
Passive coping			**2.45 [1.27, 4.72]**
**Lifetime suicide attempts**			
**Sexual minority young adults**			
Active coping	**0.57 [0.44, 0.74]**	**0.59 [0.45, 0.78]**	1.01 [0.74, 1.38]
Avoidant coping		1.14 [0.83, 1.56]	**0.61 [0.42, 0.87]**
Passive coping			**3.83 [2.77, 5.30]**
**Gender minority young adults**			
Active coping	**0.58 [0.38, 0.87]**	**0.60 [0.40, 0.92]**	0.76 [0.49, 1.19]
Avoidant coping		1.23 [0.81, 1.86]	0.84 [0.51, 1.37]
Passive coping			**2.14 [1.29, 3.57]**
**Past‐year suicidal ideation**			
**Sexual minority young adults**			
Active coping	**0.29 [0.22, 0.37]**	**0.33 [0.26, 0.43]**	**0.61 [0.46, 0.81]**
Avoidant coping		**1.92 [1.45, 2.55]**	0.94 [0.68, 1.30]
Passive coping			**5.32 [3.90, 7.24]**
**Gender minority young adults**			
Active coping	**0.54 [0.36, 0.81]**	**0.62 [0.41, 0.94]**	0.93 [0.58, 1.48]
Avoidant coping		**2.01 [1.31, 3.10]**	1.10 [0.66, 1.84]
Passive coping			**3.47 [2.02, 5.96]**

*Note*: Controlling for sex assigned at birth and age. Bold estimates are significant, *p* < 0.05.

Abbreviation: aOR, adjusted odds ratio.

Results from step 2 (avoidant coping was added) showed that for both sexual minority and gender minority participants, a higher level of active coping was still associated with a lower likelihood of lifetime and past‐year suicidal ideation and lifetime suicide attempts. For sexual minority participants, a higher level of avoidant coping was associated with a higher likelihood of lifetime and past‐year suicidal ideation. For gender minority participants, a higher level of avoidant coping was only associated with a higher likelihood of past‐year suicidal ideation.

Results from step 3 (passive coping was added) showed that for both sexual minority and gender minority participants, a higher level of passive coping was associated with a higher likelihood of lifetime and past‐year suicidal ideation and lifetime suicide attempts. However, with passive coping included in the model, active coping only remained significantly associated with past‐year suicidal ideation among sexual minority participants. Notably, for sexual minority participants, higher levels of avoidant coping were associated with a *lower* likelihood of lifetime suicidal ideation and attempts.

Regarding past‐year suicide attempts, results of the manova analyses are shown in Table 5. Sexual and gender minority participants who reported past‐year suicide attempts had a higher mean level of passive coping and a lower mean level of active coping than participants who did not report past‐year suicide attempts. In addition, sexual minority participants who reported past‐year suicide attempts had a higher mean level of avoidant coping.

### Interactions effects between minority stressors and suicidality

**FIGURE 1:**
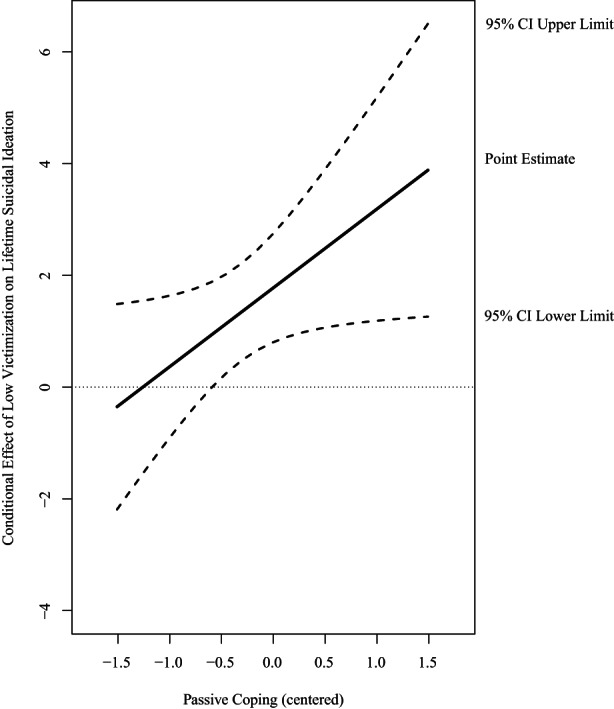
Conditional effect of low victimization on lifetime suicidal ideation at values of the moderator passive coping expressed in log odds among gender minority young adults. Abbreviation: CI, confidence interval.

For sexual minority participants, interaction effects between minority stressors and coping styles in relation to lifetime suicidal ideation were non‐significant. For gender minority participants, analyses including interaction effects between minority stressors and coping styles demonstrated that one out of 27 interaction terms were significant (see Table S1). Passive coping significantly moderated the association between low victimization and lifetime suicide ideation (odds ratio (OR) = 4.10; 95% confidence interval (CI) [1.05, 16.05]). Passive coping enhanced the association between victimization and lifetime suicidal ideation. When passive coping was low (*b* = 0.88, SE = 0.49, *p* = 0.075), there was a non‐significant association between low victimization and suicidal ideation. When passive coping was average (*b* = 1.78, SE = 0.50, *p* < 0.001) or high (*b* = 2.67, SE = 0.80, *p* < 0.001), there was a significant association between low victimization and lifetime suicidal ideation (see Figure 1).

**FIGURE 2:**
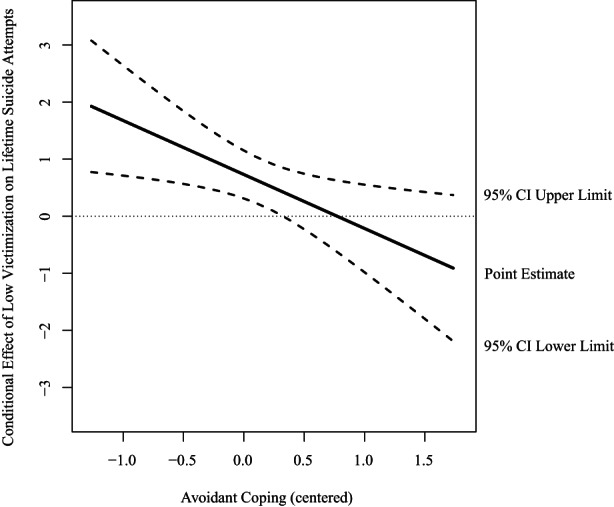
Conditional effect of low victimization on lifetime suicide attempts at values of the moderator avoidant coping expressed in log odds among sexual minority young adults. Abbreviation: CI, confidence interval.

**FIGURE 3:**
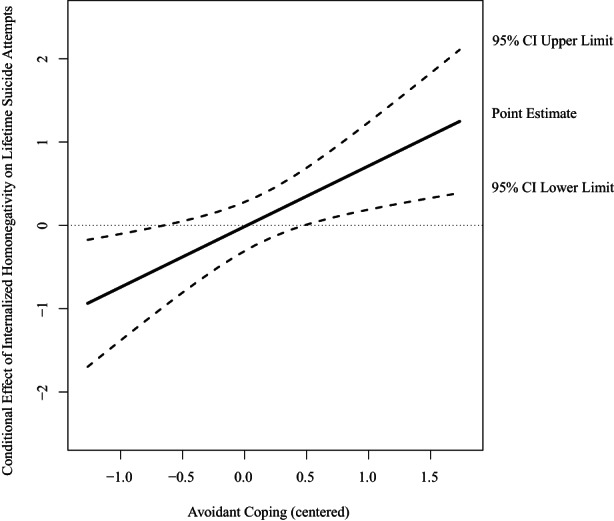
Conditional effect of internalized homonegativity on lifetime suicide attempts at values of the moderator avoidant coping expressed in log odds among sexual minority young adults. Abbreviation: CI, confidence interval.

**FIGURE 4:**
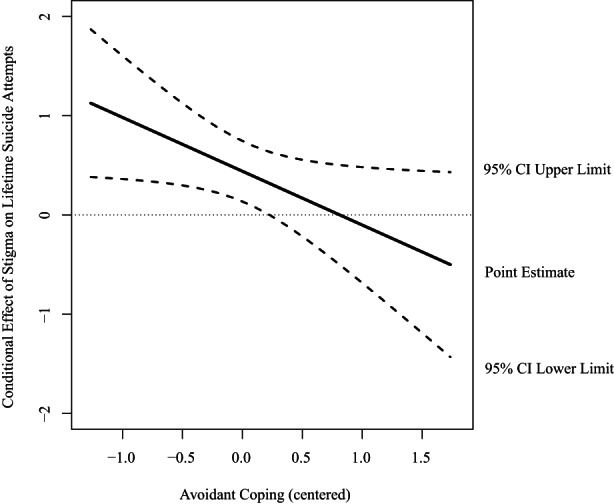
Conditional effect of stigma consciousness on lifetime suicide attempts at values of the moderator avoidant coping expressed in log odds among sexual minority young adults. Abbreviation: CI, confidence interval.

Regarding lifetime suicide attempts, for sexual minority participants, results demonstrated that three out of 36 interaction terms were significant (see Table S2). Avoidant coping significantly moderated the associations between low victimization (OR = 0.39; 95% CI [0.18, 0.84]), internalized homonegativity (OR = 2.07; 95% CI [1.25, 3.43]), stigma (OR = 0.58; 95% CI [0.35, 0.98]), and lifetime suicide attempts. More specifically, avoidant coping buffered the association between victimization and lifetime suicide attempts. When avoidant coping was low (*b* = 1.23, SE = 0.33, *p* < 0.001) or average (*b* = 0.73, SE = 0.21, *p* < 0.001), the association between low victimization (versus no) and lifetime suicide attempts was significant. When avoidant coping was high (*b* = 0.24, SE = 0.25 *p* = 0.341), the association between low victimization and lifetime suicide attempts was non‐significant (see Figure 2). Further, the interaction between avoidant coping and internalized homonegativity showed that avoidant coping enhanced the association. When avoidant coping was high (*b* = 0.36, SE = 0.18, *p* = 0.040), the association between internalized homonegativity and lifetime suicide attempts was significant. When avoidant coping was low (*b* = −0.40, SE = 0.22, *p* = 0.076) or average (*b* = −0.02, SE = 0.15, *p* = 0.908), there was a non‐significant association between internalized homonegativity and lifetime suicide attempts (see Figure 3). Last, the interaction between coping and stigma consciousness showed that avoidant coping buffered the association. When avoidant coping was low (*b* = 0.73, SE = 0.22, *p* < 0.001) or average (*b* = 0.44, SE = 0.15, *p* = 0.004), the association between stigma and lifetime suicide attempts was significant. When avoidant coping was high (*b* = 0.16, SE = 0.20, *p* = 0.426), there was a non‐significant association between stigma and lifetime suicide attempts (see Figure 4). For gender minority participants, interaction effects between minority stressors and coping styles were non‐significant. Regarding past‐year suicidal ideation, for both sexual and gender minority participants interaction effects between minority stressors and coping styles were non‐significant.

## DISCUSSION

In line with our hypotheses and previous research (de Lange et al., 2022; Hatchel et al., 2019; Kuper et al., 2018; Testa et al., 2017), we found that minority stressors were associated with suicidality among SGM young adults. Among sexual minority young adults, victimization and stigma consciousness were associated with (past‐year) suicidal ideation and lifetime suicide attempts. Internalized homonegativity was only associated with suicidal ideation, and not with lifetime suicide attempts. Among gender minority young adults, victimization and stigma consciousness were also associated with (past‐year) suicidal ideation, but only high victimization was associated with lifetime suicide attempts. Both sexual and gender minority participants who were victimized once or more per month were around 3.5 times more likely to report ever having attempted suicide. The integrated motivational‐volitional model of suicidal behavior (IVM) (O'Connor & Kirtley, 2018) suggests that suicidal behavior may be dependent on several volitional factors. Possibly, there is no direct relationship between some minority stressors and suicidal behavior because this association is dependent on other volitional factors such as impulsivity or past suicidal behavior.

Next, partly in line with our hypotheses and previous research (Benatov et al., 2020; Ong & Thompson, 2019), our results showed that active coping was associated with a lower likelihood of suicidality among SGM young adults, even when avoidant coping was added to the model. However, when passive coping was added to the model, active coping was no longer associated with suicidality. Avoidant coping was associated with a higher likelihood of lifetime and past‐year suicidal ideation among sexual minority young adults, and with a higher likelihood of past‐year suicidal ideation among gender minority young adults. Further, passive coping was associated with a higher likelihood of suicidality among both sexual and gender minority young adults, even when active and avoidant coping were included in the model. Cognitive processes related to passive coping are similar to rumination, which is an important contributor to suicidal ideation in the IVM model (O'Connor & Kirtley, 2018). A meta‐analytic review on rumination and suicidality underlined that rumination can be an important risk factor for suicidal ideation (Rogers & Joiner, 2017).

In addition, when the three coping styles were assessed together in one model, an unexpected result emerged—a higher level of avoidant coping was associated with a lower likelihood of lifetime suicidal ideation among sexual minority participants. While avoidant coping was associated with a higher likelihood of lifetime suicidal ideation when only active and avoidant coping were included in the model. We do not have a clear explanation for this event. This could be a statistical artifact or point to an interaction between different forms of coping. Unfortunately, we did not have the statistical power to assess interactions between different coping styles. Therefore, we urge future research to examine the association between different forms of coping and interactions between them, in relation to suicidality.

The findings of the current study underscore the importance of increasing active coping and decreasing avoidant and passive coping in young SGM individuals. In particular, passive coping is an important maladaptive coping style for SGM young adults and should be targeted in prevention and intervention efforts for SGM young adults.

We also examined whether coping mitigated or exacerbated the associations between minority stress and suicidality. The results of this study demonstrated that an average or relatively high level of passive coping enhanced the association between low victimization (compared with no victimization) and suicidal ideation for gender minority young adults. Among sexual minority young adults, avoidant coping buffered the relations between victimization, stigma, and lifetime suicide attempts. In contrast, avoidant coping enhanced the association between internalized homonegativity and suicide attempts. As previously proposed (D'haese et al., 2016; White Hughto et al., 2017), these mixed results may be best explained by the context of victimization. Individuals who regularly experience LGBT‐related victimization may avoid certain situations or individuals, and as a result, their mental health is temporarily less impacted. Avoidant coping therefore seems to be protective in the short term, but it is not clear how this affects mental health in the long term. Moreover, avoiding situations or individuals may also limit opportunities for affirmation of one's identity, and links between internalized homonegativity and poorer mental health may remain intact. Moreover, previous studies found that internalized homophobia was related to higher levels of maladaptive coping, which in turn was related to higher levels of psychological distress among sexual minority women (Kaysen et al., 2014; Szymanski et al., 2014). Possibly, SGM young adults develop maladaptive coping styles in response to experiencing minority stress.

Finally, in line with our hypotheses, among SGM young adults who attempted suicide in the past year, we found higher rates of victimization, higher levels of internalized homonegativity, stigma, avoidant coping, and passive coping, and lower levels of active coping. Among gender minority individuals who reported a past‐year suicide attempt, we found higher levels of stigma consciousness and passive coping, and lower levels of active coping. No mean level differences for avoidant coping were found, and no association was found for victimization. These findings underline the importance of further examining the role of coping in individuals with recent suicide attempts.

### Strengths, limitations, and future research

To the best of our knowledge, this is one of the first studies to assess coping styles as moderators of the associations between minority stress and suicidality among SGM young adults. In addition, we assessed our hypotheses among sexual minority and gender minority individuals separately, which allowed for a comprehensive perspective of their unique experiences with minority stress.

This study also has some limitations. First, because of the cross‐sectional design, we were unable to make statements about temporal effects. Although we assume that SGM young adults apply their coping styles to cope with potential minority stress experiences, it could be that minority stress experiences affect the coping styles that individuals develop and utilize. In addition, it is unclear how coping styles relate to short‐term or long‐term mental health outcomes, including suicidal ideation and attempts in adulthood. Longitudinal research, across multiple years, is necessary to study the development of both minority stress, coping styles, and suicidality. With such a study, we would be able to tease apart the temporal order of these concepts and better understand what the most optimal time to intervene is. Second, suicidal ideation and suicide attempts were assessed with single‐item measures. Although research has found preliminary support for the validity of such items to measure suicidality (May & Klonsky, 2011), there is also research showing that single‐item measures of suicidality could lead to misclassification (Hom et al., 2016; Millner et al., 2015). Thus, participants in our study may have been classified as having suicidal ideation or having attempted suicide, while with a multiple‐item measure they would not be classified as such. Nevertheless, one‐item measures do indicate that participants are at risk for suicidality (Hom et al., 2016). Further, our aim was not to assess the prevalence of suicidality among SGM young adults, nor was it used as a screening tool in clinical settings. It remains important that future research assesses the validity of single‐item and multiple‐item measures for examining suicidality among young people. Third, recruitment of participants was partly done via suicide prevention websites to collect sufficient data on the outcome of suicide attempts. The findings of the current study may therefore not be generalizable to the overall SGM young adult population in Flanders and the Netherlands. Fourth, due to power limitations, we were unable to assess past‐year suicide attempts in our predictive models. Univariate and multivariate analyses did give some insight into differences in minority stress and coping styles regarding past‐year suicide attempts. However, these results need to be interpreted with caution, because the number of individuals who reported past‐year suicide attempts was small. Finally, for minority stress, we were limited to the assessment of internalized homonegativity—a measure of internalized trans negativity was not assessed.

For future research, we would advise the inclusion of multiple measures of minority stress, specific to a sexual or gender minority group. In addition, it would be informative for interventions to investigate SGM‐specific coping styles in relation to suicidality. For example, Meyer (2003) suggests that a connection with the LGB community can protect against poor mental health, and in a previous study LGB‐specific coping was indeed associated with less depressive symptoms (Toomey et al., 2018).

### Clinical implications

Insight from this research should inform (preventive) intervention efforts and support for SGM young adults with (or at risk for) suicidal ideation. Our findings suggest that increasing adaptive coping styles and decreasing maladaptive coping styles could decrease suicidality in SMG young adults. Some existing interventions target coping skills in SGM young individuals (Coulter et al., 2019; Craig et al., 2019), but these do not specifically target suicidality and/or exclude participants with active suicidal ideation (Craig et al., 2019). Moreover, a meta‐analytic review on suicide prevention interventions for young people showed that few interventions focused on SGM young people (Robinson et al., 2018), while a study also suggests that SGM young people search for SGM affirming crisis services (Goldbach et al., 2019). Taken together, mental health services and interventions must be SGM affirming and target coping skills and suicidality.

In addition, safety‐planning interventions, which are increasingly used as an effective prevention effort by mental healthcare professionals, can include coping strategies that individuals can use in times of crisis (Stanley & Brown, 2012). As such, safety planning interventions with coping features are a promising tool for suicide prevention among SGM young adults as well (Nuij et al., 2021; Stanley & Brown, 2012).

## CONCLUSION

This study addressed a gap in the literature by examining the association between minority stress, coping styles, and suicidal ideation and attempts among SGM young adults. Our findings underline the importance of coping styles in relation to suicidal ideation and attempts. Furthermore, our findings highlight the need for further research into coping styles and suicidality to optimize suicide prevention interventions and mental healthcare for SGM young adults with suicidal ideation.

## Funding information

This study was supported by ZonMw, the Netherlands Organization for Health Research and Development [grant number 531 004005, 2017]. Jo Robinson is supported by NHMRC Career Development Fellowship (APP1142348).

## CONFLICT OF INTEREST

The authors declare no conflicts of interest.

## Ethics approval statement

Ethics approval was given by the ethics committee of the Department of Pedagogy and Educational Sciences of the University of Groningen, and the Ghent University Hospital.

## Supporting information


Table S1

Table S2

## Data Availability

The research data are not shared.
