# Supplementary material for: Moderating role of coping in the association between minority stress and suicidal ideation and suicide attempts among sexual and gender minority young adults
Source: Suicide Life Threat Behav. 2022 Sep 2;52(6):1178–92. doi: 10.1111/sltb.12913 (PMC10946947; doi:10.1111/sltb.12913)
Supplement: Supplementary file 1 — Table S1 [file SLTB-52-1178-s001.docx]

|  | Lifetime suicidal ideation | Lifetime suicide attempts | Past-year suicidal ideation |
| --- | --- | --- | --- |
|  | aOR [95% CI] | aOR [95% CI] | aOR [95% CI] |
| Active coping × low  victimization | 0.38 [0.09, 1.54] | 0.95 [0.24, 3.85] | 1.41 [0.37, 5.39] |
| Active coping × high  victimization | 0.90 [0.19, 4.31] | 1.79 [0.42, 7.57] | 1.56 [0.37, 6.63] |
| Active coping × stigma  consciousness | 0.70 [0.29, 1.68] | 1.07 [0.57, 2.04] | 0.92 [0.47, 1.82] |
| Avoidant coping × low  victimization | 0.90 [0.24, 3.43] | 0.98 [0.30, 3.17] | 0.67 [0.20, 2.26] |
| Avoidant coping × high  victimization | 1.10 [0.23, 5.29] | 1.02 [0.28, 3.72] | 0.57 [0.15, 2.17] |
| Avoidant coping ×  stigma consciousness | 0.78 [0.35, 1.77] | 1.02 [0.55. 1.90] | 0.86 [0.43, 1.73] |
| Passive coping × low  victimization | **4.10 [1.05, 16.05]** | 2.23 [0.71, 6.95] | 1.39 [0.41, 4.65] |
| Passive coping × high  victimization | 1.67 [0.40, 7.14] | 1.86 [0.54, 6.37] | 0.90 [0.25, 3.30] |
| Passive coping ×  stigma consciousness | 1.31 [0.60, 2.87] | 0.85 [0.44, 1.63] | 0.66 [0.32, 1.37] |

Table S1. Results of moderation analyses for gender minority young adults

*Note.* All three coping styles were assessed separately. Controlling for sex assigned at birth and age. Bold estimates are significant, p < .05. aOR = adjusted odds ratio; CI = confidence interval; low victimization = sometimes; high victimization = once per month or more.
