# Supplementary material for: Moderating role of coping in the association between minority stress and suicidal ideation and suicide attempts among sexual and gender minority young adults
Source: Suicide Life Threat Behav. 2022 Sep 2;52(6):1178–92. doi: 10.1111/sltb.12913 (PMC10946947; doi:10.1111/sltb.12913)
Supplement: Supplementary file 2 — Table S2 [file SLTB-52-1178-s002.docx]

Table S2. Results of moderation analyses for sexual minority young adults

|  | Lifetime suicidal ideation | Lifetime suicide attempts | Past-year suicidal ideation |
| --- | --- | --- | --- |
|  | aOR [95% CI] | aOR [95% CI] | aOR [95% CI] |
| Active coping × low  victimization | 1.24 [0.77, 2.01] | 0.87 [0.46, 1.65] | 1.33 [0.75, 2.37] |
| Active coping × high  victimization | 1.82 [0.90, 3.69] | 0.84 [0.38, 1.86] | 1.25 [0.56, 2.82] |
| Active coping × internalized  homonegativity | 0.97 [ 0.67, 1.41] | 0.80 [0.52, 1.24] | 1.00 [0.65, 1.56] |
| Active coping × stigma  consciousness | 0.86 [0.57, 1.28] | 1.14 0.70, 1.84] | 0.78 [0.48, 1.26] |
| Avoidant coping × low  victimization | 0.82 [0.47, 1.45] | **0.39 [0.18, 0.84]** | 0.73 [0.39, 1.36] |
| Avoidant coping × high  victimization | 0.67 [0.30, 2.05] | 0.55 [0.22, 1.34] | 1.07 [0.46, 2.51] |
| Avoidant coping ×  internalized homonegativity | 1.30 [0.82, 2.05] | **2.07 [1.25, 3.43]** | 0.89 [0.54, 1.45] |
| Avoidant coping ×  stigma consciousness | 0.79 [0.49, 1.26] | **0.58 [0.35, 0.98]** | 1.06 [0.64, 1.77] |
| Passive coping × low  victimization | 1.00 [0.54, 1.85] | 0.91 [0.49, 1.72] | 0.79 [0.42, 1.48] |
| Passive coping × high  victimization | 0.55 [0.25, 1.24] | 1.27 [0.57, 2.84] | 0.57 [0.25, 1.30] |
| Passive coping ×  internalized homonegativity | 1.66 [1.01, 2.74] | 1.01 [0.65, 1.58] | 0.97 [0.60, 1.57] |
| Passive coping ×  stigma consciousness | 0.95 [0.59, 1.55] | 0.81 [0.51, 1.28] | 1.06 [0.65, 1.72] |

*Note.* All three coping styles were assessed separately. Controlling for sex assigned at birth and age. Bold estimates are significant, p < .05. aOR = adjusted odds ratio; CI = confidence interval; low victimization = sometimes; high victimization = once per month or more.
